# Supplementary material for: A transcriptome based molecular classification scheme for cholangiocarcinoma and subtype-derived prognostic biomarker
Source: Nat Commun. 2024 Jan 11;15:484. doi: 10.1038/s41467-024-44748-8 (PMC10784309; doi:10.1038/s41467-024-44748-8)
Supplement: Supplementary file 1 — supplementary information [file 41467_2024_44748_MOESM1_ESM.pdf]

A transcriptome based molecular classification scheme for cholangiocarcinoma and subtype derived prognostic biomarker

Authors

Zhongqi Fan[1,#], Xinchen Zou[2,#], Guangyi Wang[1], Yahui Liu[1], Yanfang Jiang[3], Haoyan Wang[2], Ping Zhang[1], Feng Wei[1], Xiaohong Du[1], Meng Wang[1], Xiaodong Sun[1], Bai Ji[1], Xintong Hu[3], Liguo Chen[3], Peiwen Zhou[3], Duo Wang[3], Jing Bai[2], Xiao Xiao[4], Lijiao Zuo[2], Xuefeng Xia[2], Xin Yi[2,5], Guoyue Lv[1,*]

[1]Department of Hepatobiliary and Pancreatic Surgery, General Surgery Center, First Hospital of Jilin University, Changchun, China; [2]Geneplus-Beijing Institute, 9th Floor, No.6 Building, Peking University Medical Industrial Park, Zhongguancun Life Science Park, Beijing, China; [3]Genetic Diagnosis Center, The First Hospital of Jilin University, Changchun, China; [4]Geneplus-Shenzhen, No.14 Zhongxing Road, Pingshan District, Shenzhen, China; [5]School of Computer Science and Technology, Xi'an Jiaotong University, Xi'an, China;

[#]These authors contributed equally as co–first authors of this article.

*Corresponding author

Contents

Supplementary Figures: 16; pages 2-29.

Supplementary Tables: 10; pages 30-41.

Supplementary Figures

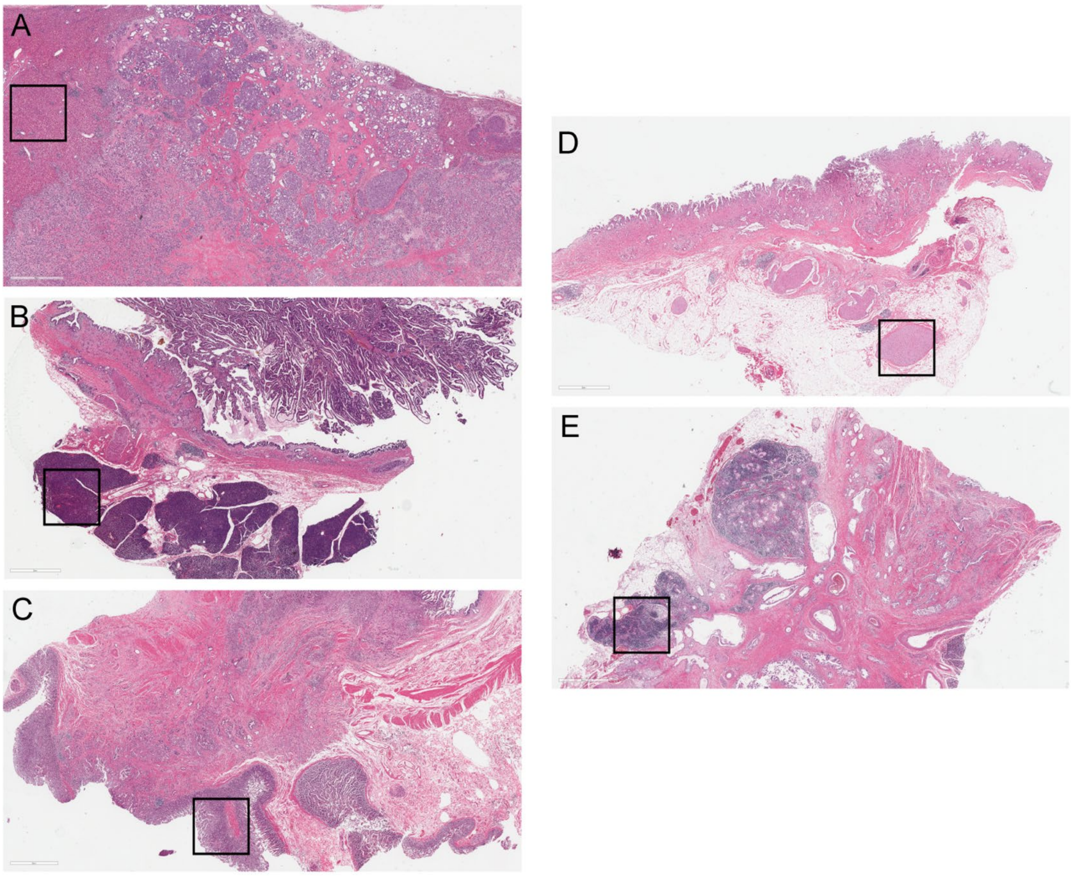

Supplementary Fig. 1. Normal tissue contamination of CCA tumor samples. Representative images of H&E staining of hepatic (**A**), pancreatic (**B**), duodenal (**C**), neural (**D**) and lymphatic (**E**) tissues contamination. Bar, 2 mm. Black box marks the normal tissue contamination.

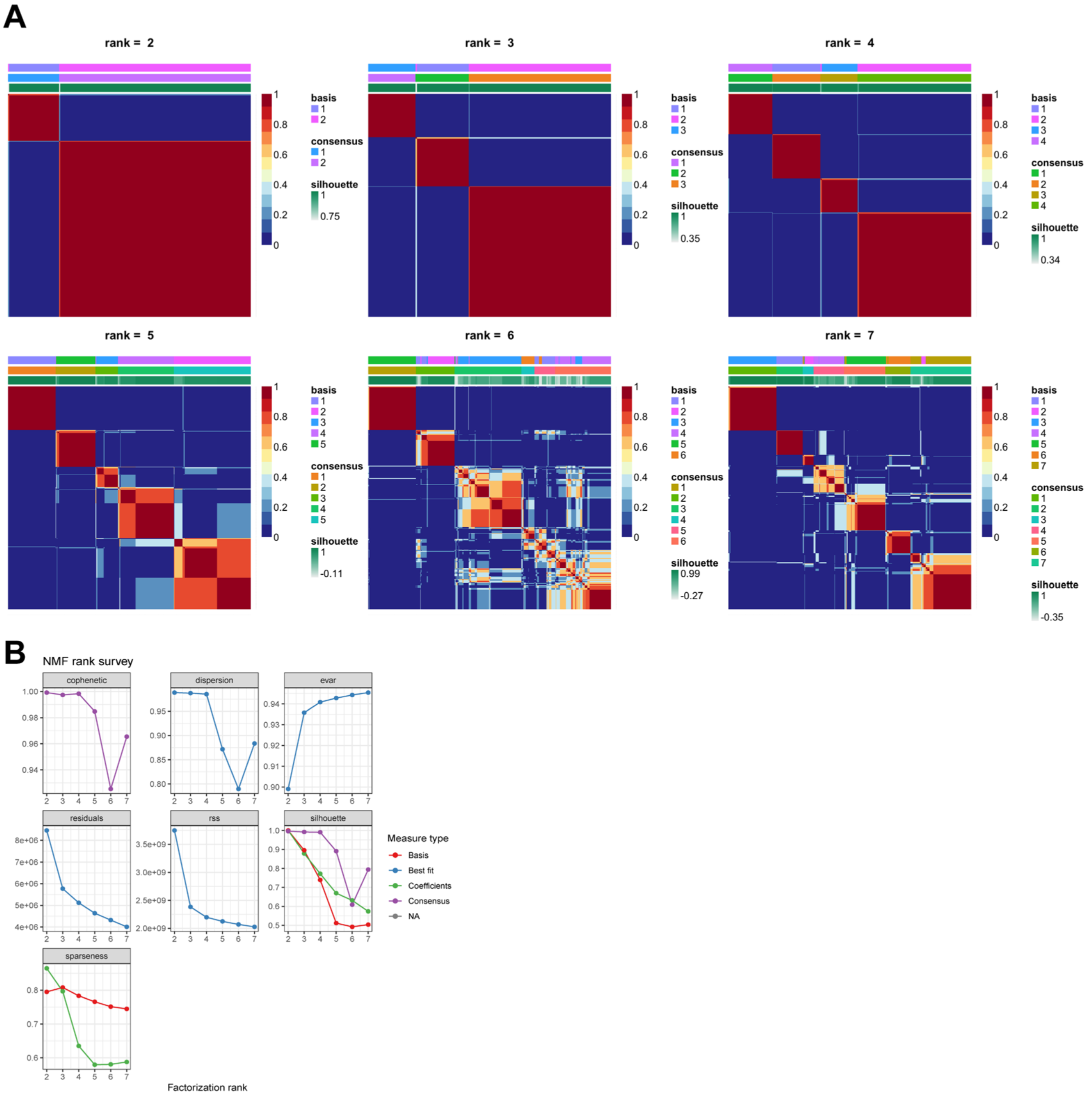

Supplementary Fig. 2. Unsupervised classification of 438 CCAs by performing non-negative matrix factorization (NMF) on transcriptomic data. (**A**) Heatmap displaying the classification solutions for k=2 to k=7 classes. (**B**) NMF rank survey representing the change of different parameter values with increasing k. The cophenetic coefficient for k=2 and k=4 was similarly high. As a solution for more classes was desired, k=4 was considered as the best solution.

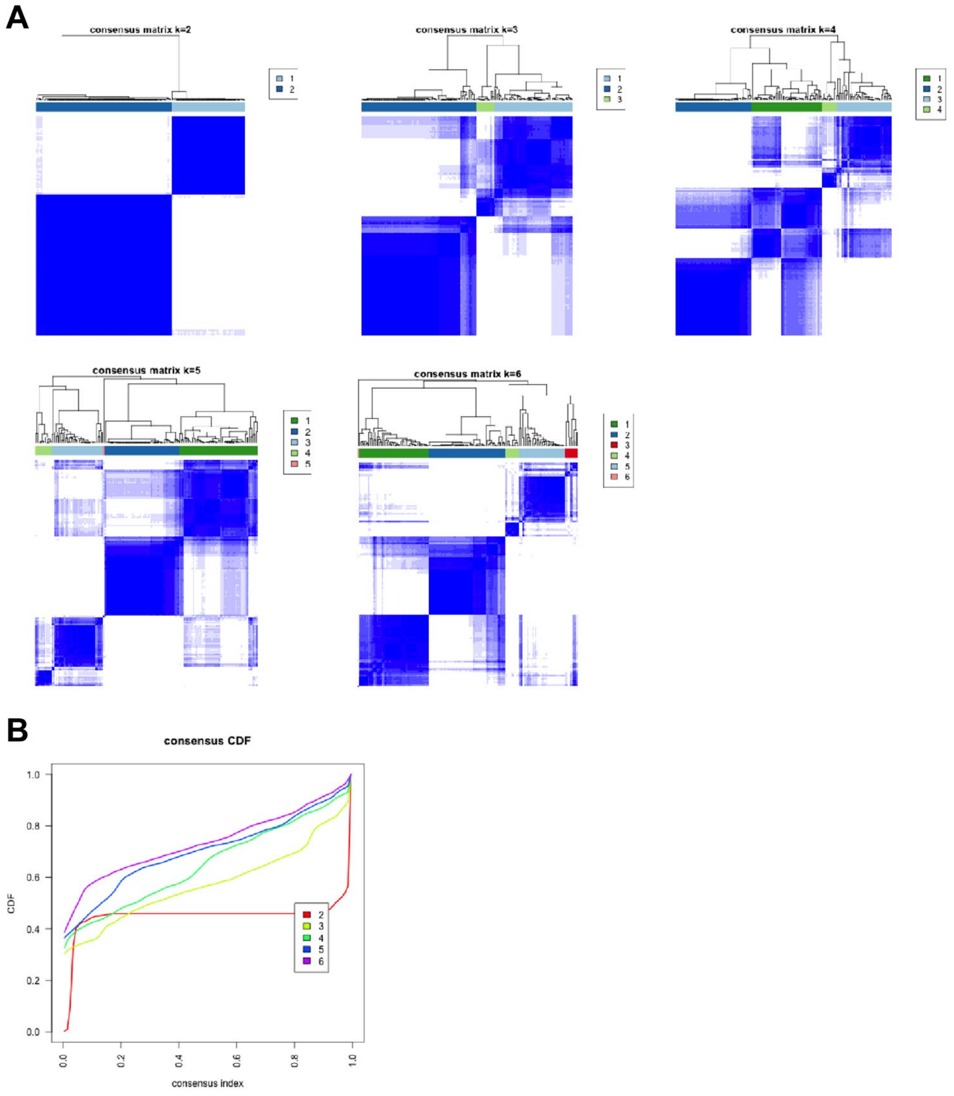

Supplementary Fig. 3. Unsupervised classification of 164 CCAs by performing consensus clustering on transcriptomic data. (**A**) Heatmap displaying the consensus matrix for *K*=2 to *K*=6 classes. (**B**) Empirical cumulative distribution functions (CDFs) corresponding to the entries of consensus matrices for *K*=2 to *K*=6. As empirical cumulative distribution function for *K*=2 was approximately constant with the increasing consensus index, *K*=2 was considered as the best solution.

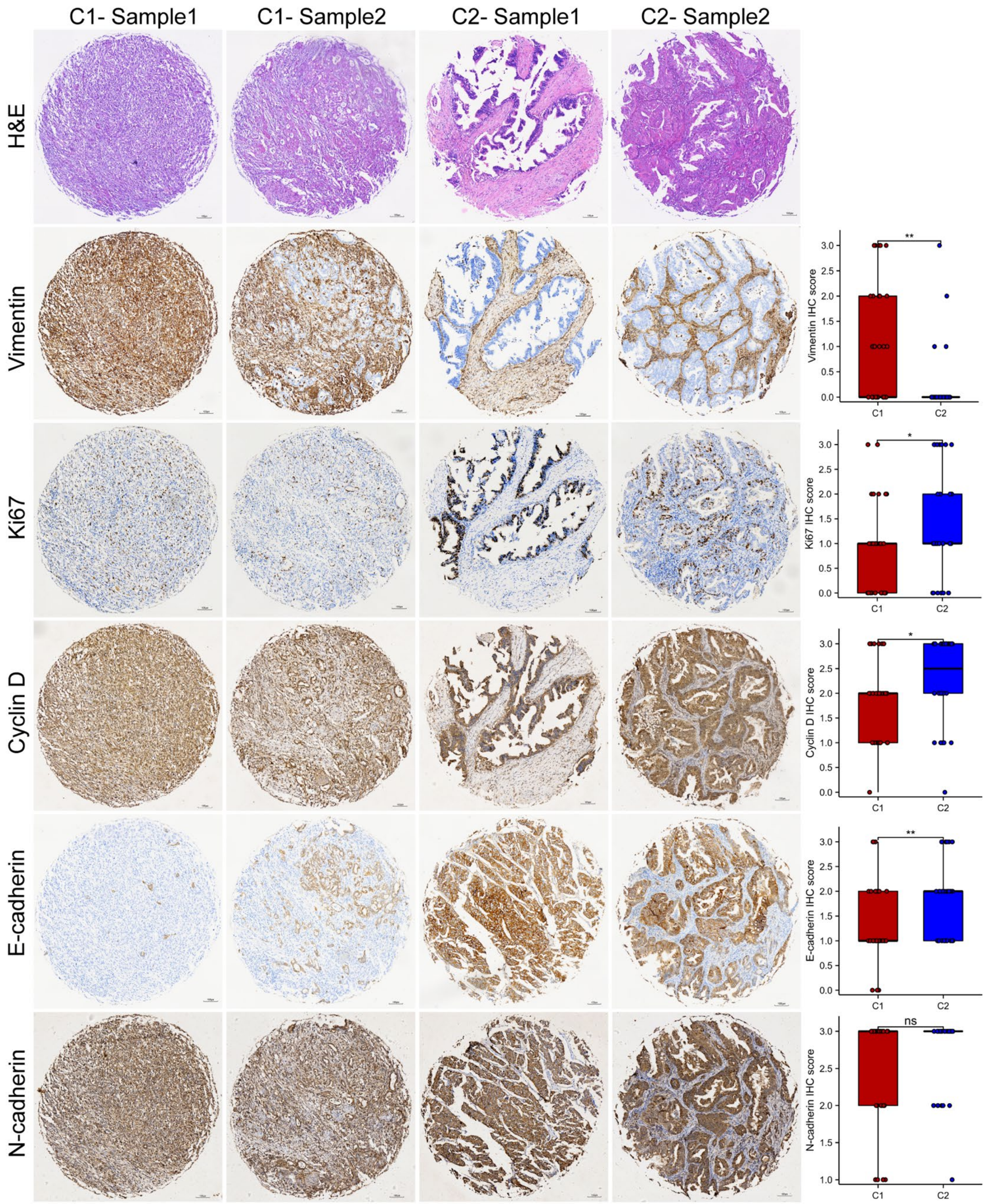

Supplementary Fig. 4. Immunofluorescence showed different EMT and proliferation characteristics of each class. IHC stanning of E-cadherin/Vimentin/N-cadherin showed the expression level of EMT core proteins. IHC stanning of Ki67 and Cyclin D showed the expression level of proliferation-associated proteins. The histogram on the right shows the

statistical results of each group staining. *P* values were calculated by two-sided Wilcoxon rank sum test. C1, n = 18; C2, n = 18.

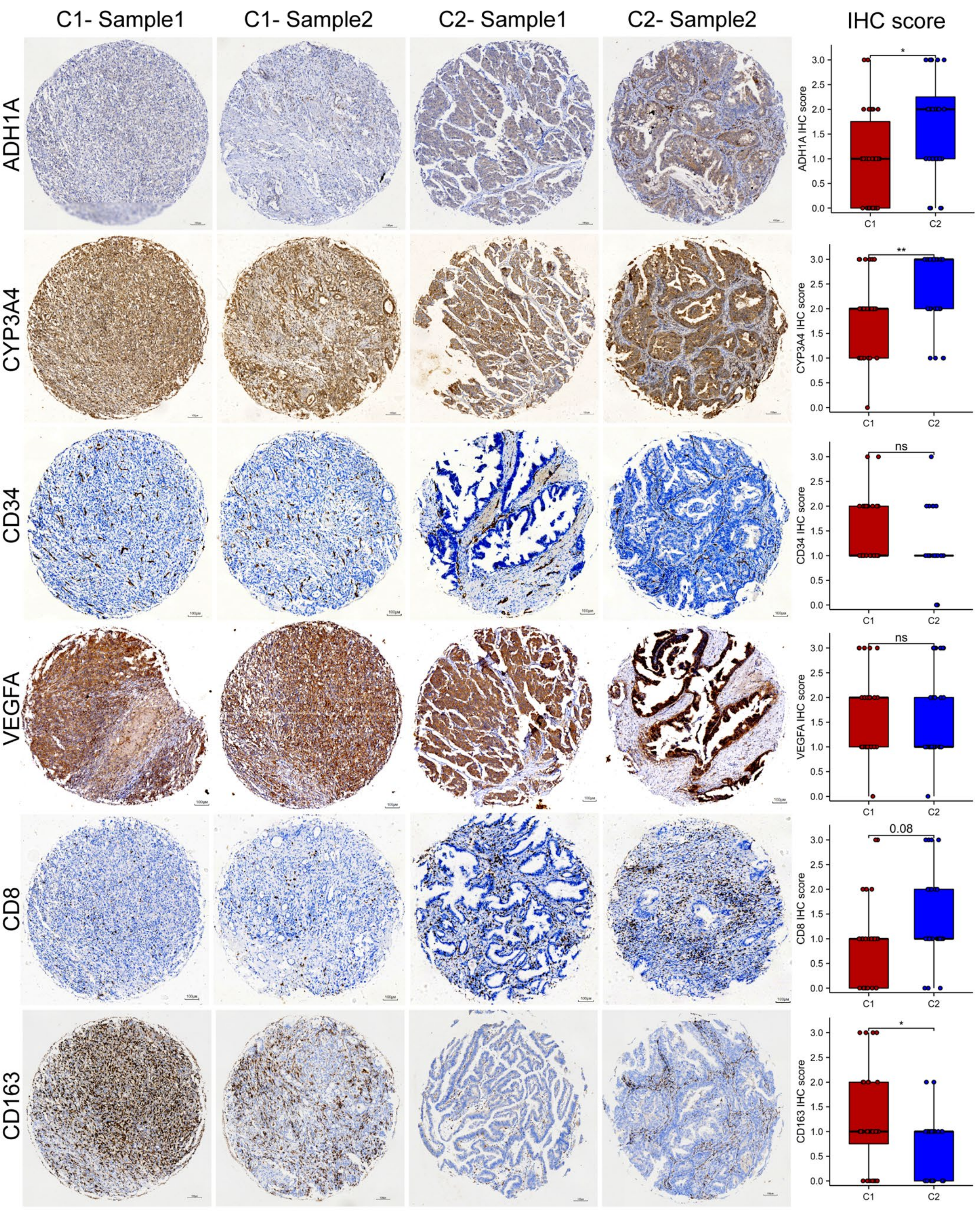

Supplementary Fig. 5. Immunofluorescence showed different metabolic/angiogenesis/immune characteristics of each class. CYP3A4 and ADH1A was used to characterize metabolic differences of each class. IHC stanning of CD34 and VEGFA showed the expression level of angiogenesis key proteins. CD8 and CD163 were used to represent the

infiltration of T cells and M2 macrophage, respectively. The histogram on the right shows the statistical results of each group staining. *P* values were calculated by two-sided Wilcoxon rank sum test. C1, n = 18; C2, n = 18.

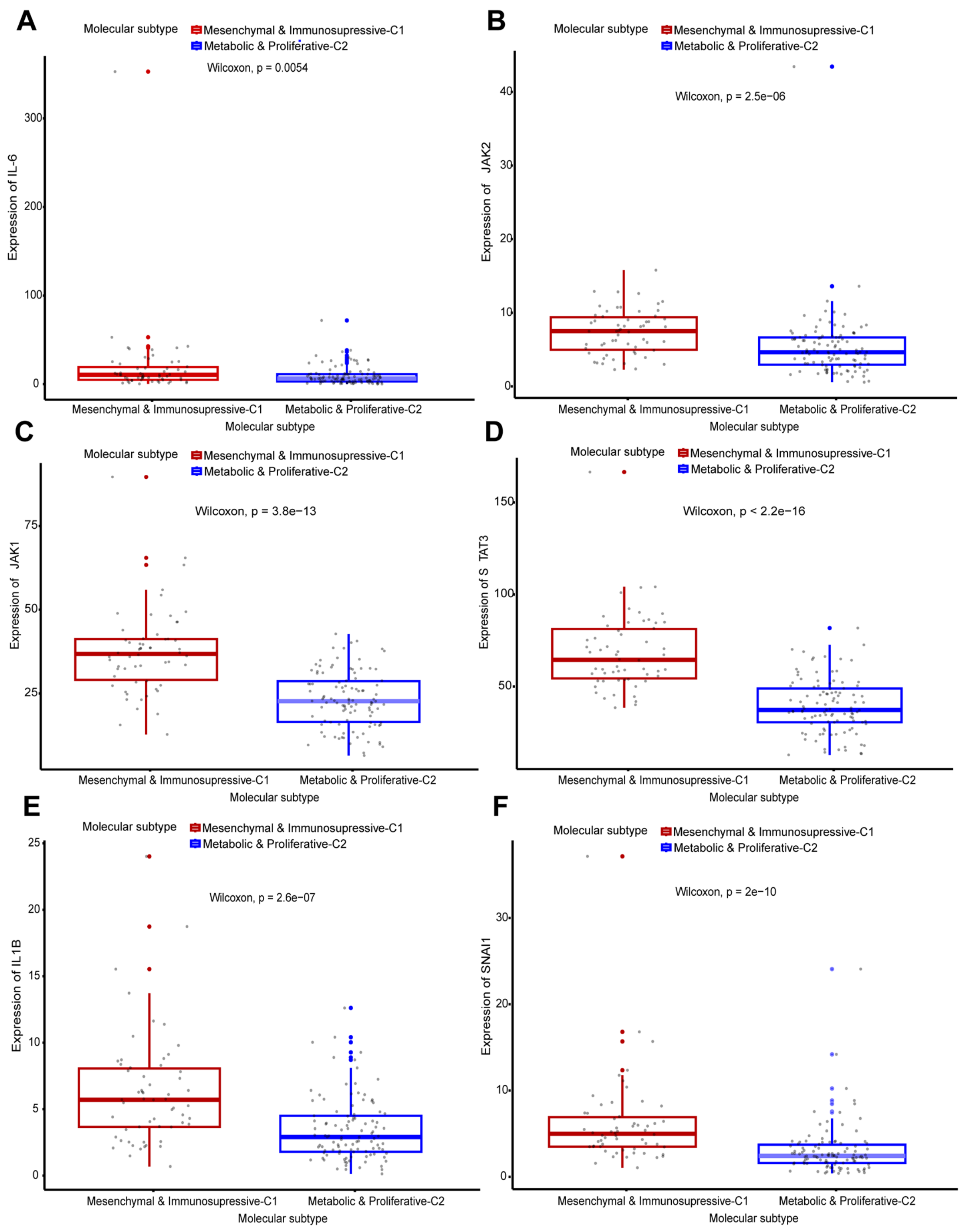

Supplementary Fig. 6. Relative RNA level of genes associated with classical oncogenic signaling pathways in each class. Box plots representing the relative RNA expression of (**A**) *IL-6*; (**B**) *JAK2*; (**C**) *JAK1*; (**D**)

STAT3; (**E**) *IL-1B*; (**F**) *SNAI1*. *P* values were calculated by two-sided Wilcoxon rank sum test. C1, n = 58; C2, n = 108.

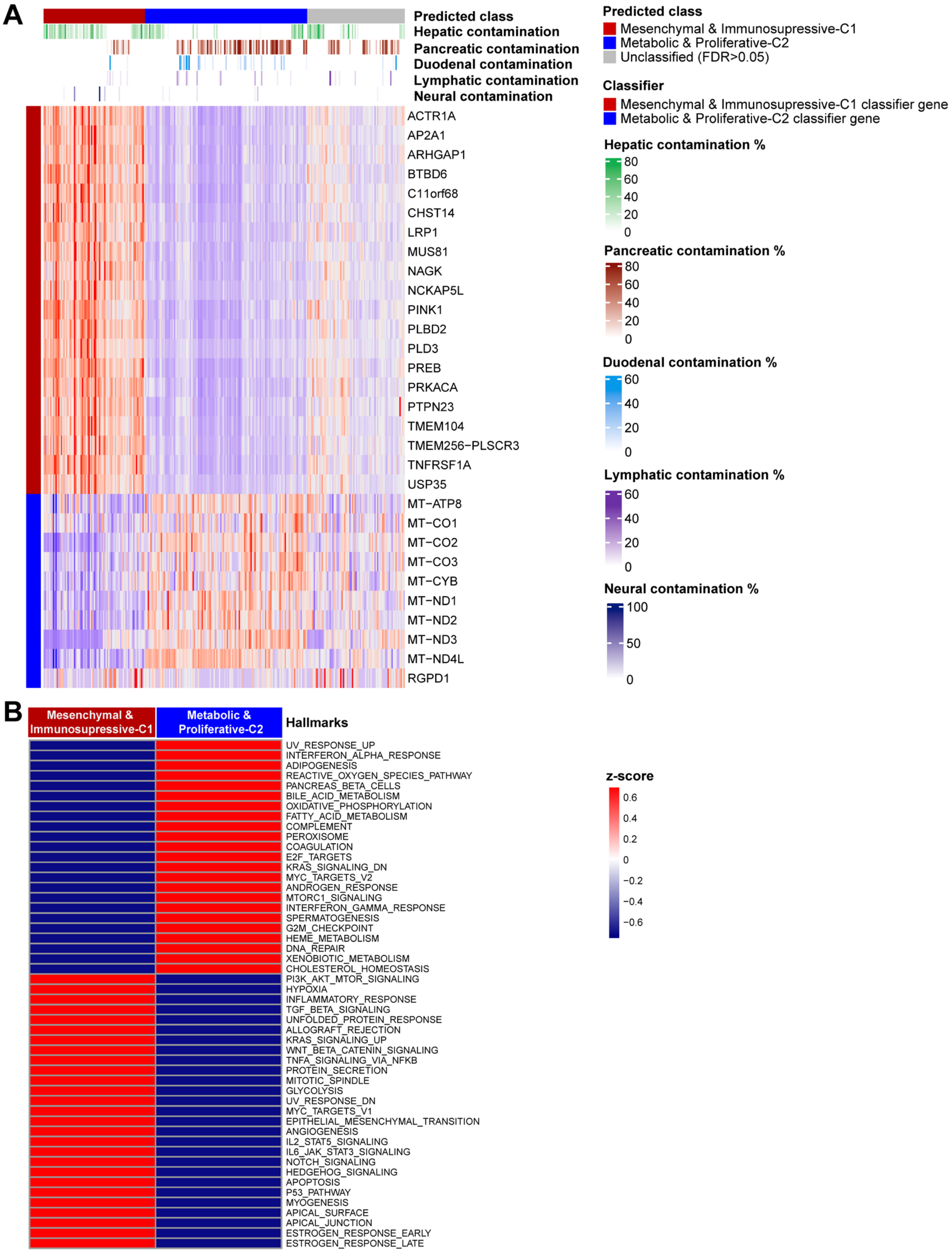

Supplementary Fig. 7. Validation of classifier and molecular classification scheme on the verification cohort. (**A**) Heatmap representing the expression of classifier genes in each sample (verification cohort, N=274). The expression levels were represented by normalized z-

scores. The molecular class was predicted by nearest template prediction (NTP) analysis. (**B**) Heatmap representing the enrichment of hallmark gene sets in molecular classes for the verification cohort. Single-sample gene set enrichment analysis (ssGSEA) was used to obtain enrichment scores, with samples from the same subtype indicated with a normalized z-score.

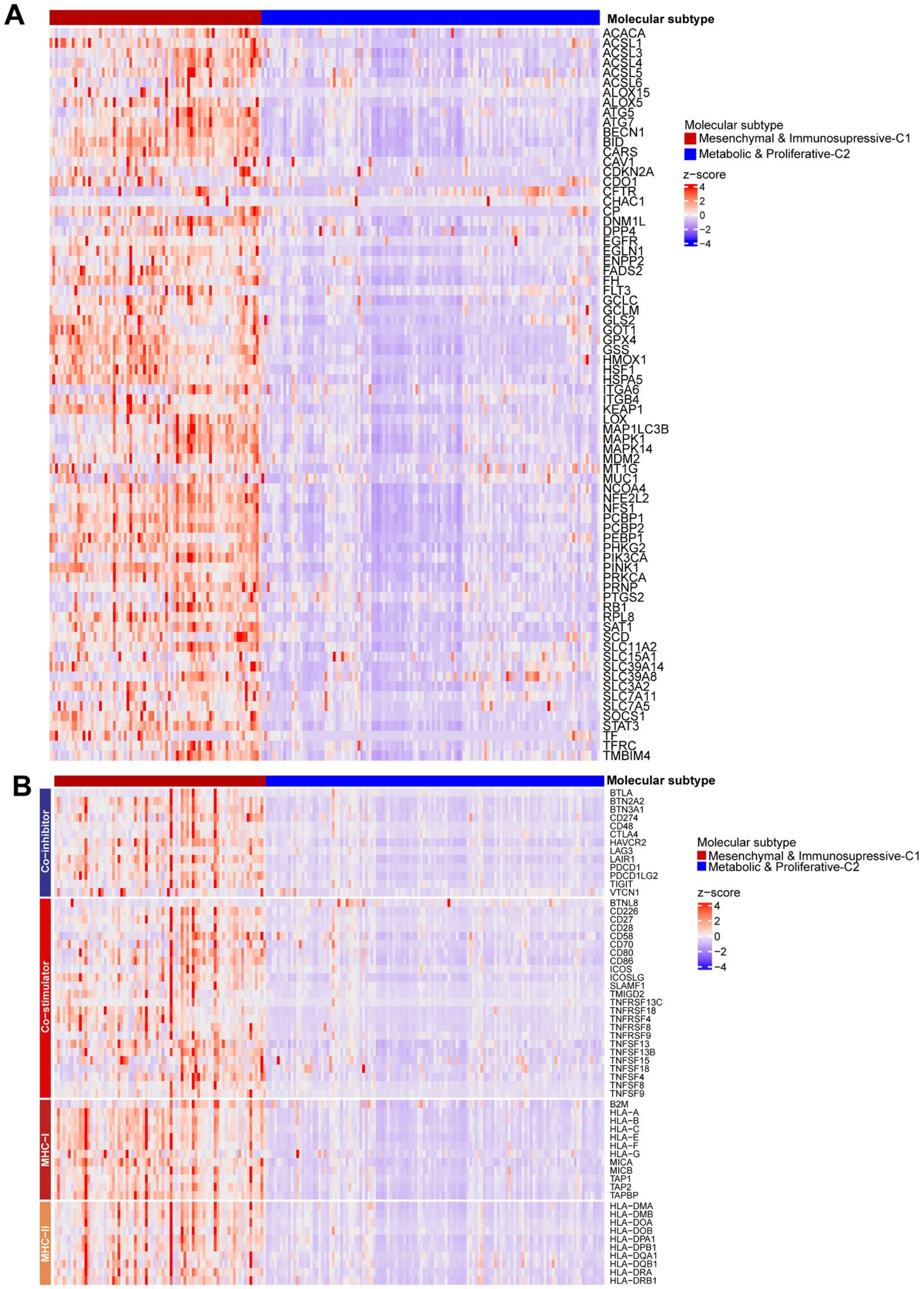

Supplementary Fig. 8. Validation of ferroptosis-related features and immune escape mechanisms of molecular classes on the verification cohort. Heatmaps displaying expression levels of (**A**) ferroptosis-related genes; and (**B**) genes encoding co-stimulators, co-inhibitors and MHC

antigens in each molecular class. The expression values were normalized and represented by z-scores. N=274.

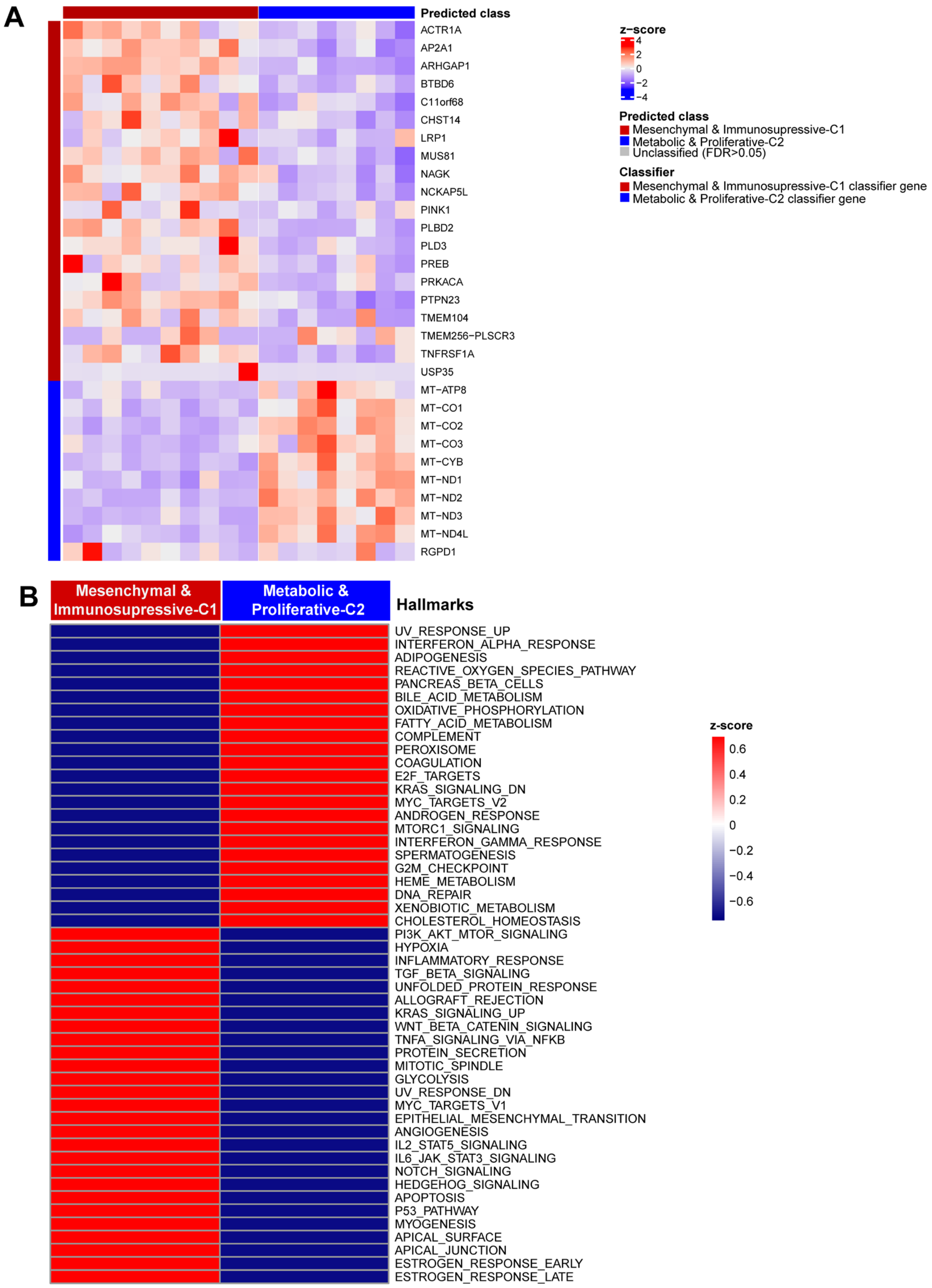

Supplementary Fig. 9. Validation of classifier and molecular classification scheme on the TCGA-CHOL samples. (**A**) Heatmap representing the expression of classifier genes in each sample (TCGA-CHOL project, N=36). The expression levels were represented by normalized z-

scores. The molecular class was predicted by nearest template prediction (NTP) analysis. (**B**) Heatmap representing the enrichment of hallmark gene sets in molecular classes for the TCGA-CHOL samples. Single-sample gene set enrichment analysis (ssGSEA) was used to obtain enrichment scores, with samples from the same subtype indicated with a normalized z-score.

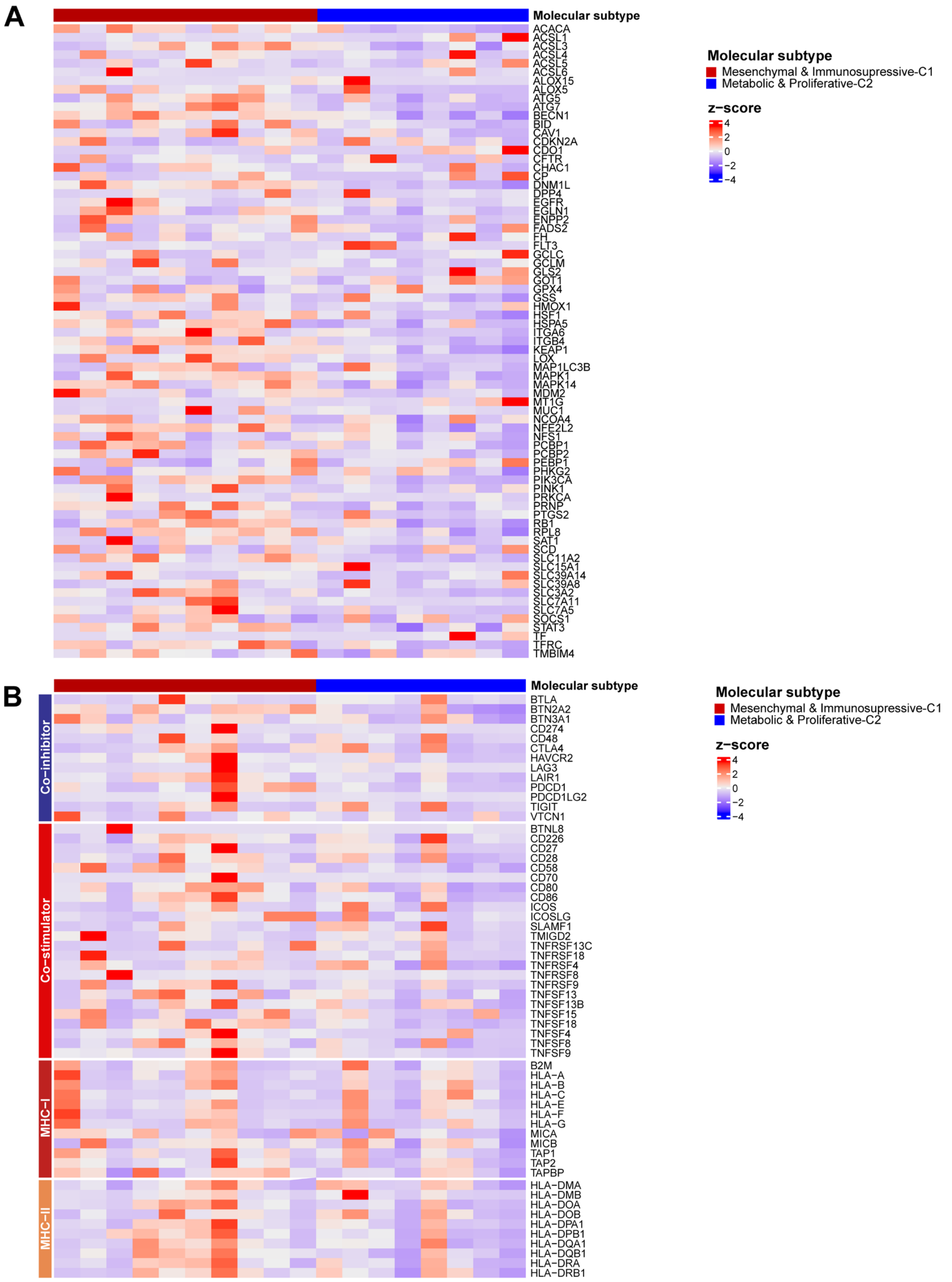

Supplementary Fig. 10. Validation of ferroptosis-related features and immune escape mechanisms of molecular classes on the TCGA-CHOL samples. Heatmaps displaying expression levels of (**A**) ferroptosis-related genes; and (**B**) genes encoding co-stimulators, co-inhibitors and MHC

antigens in each molecular class. The expression values were normalized and represented by z-scores.

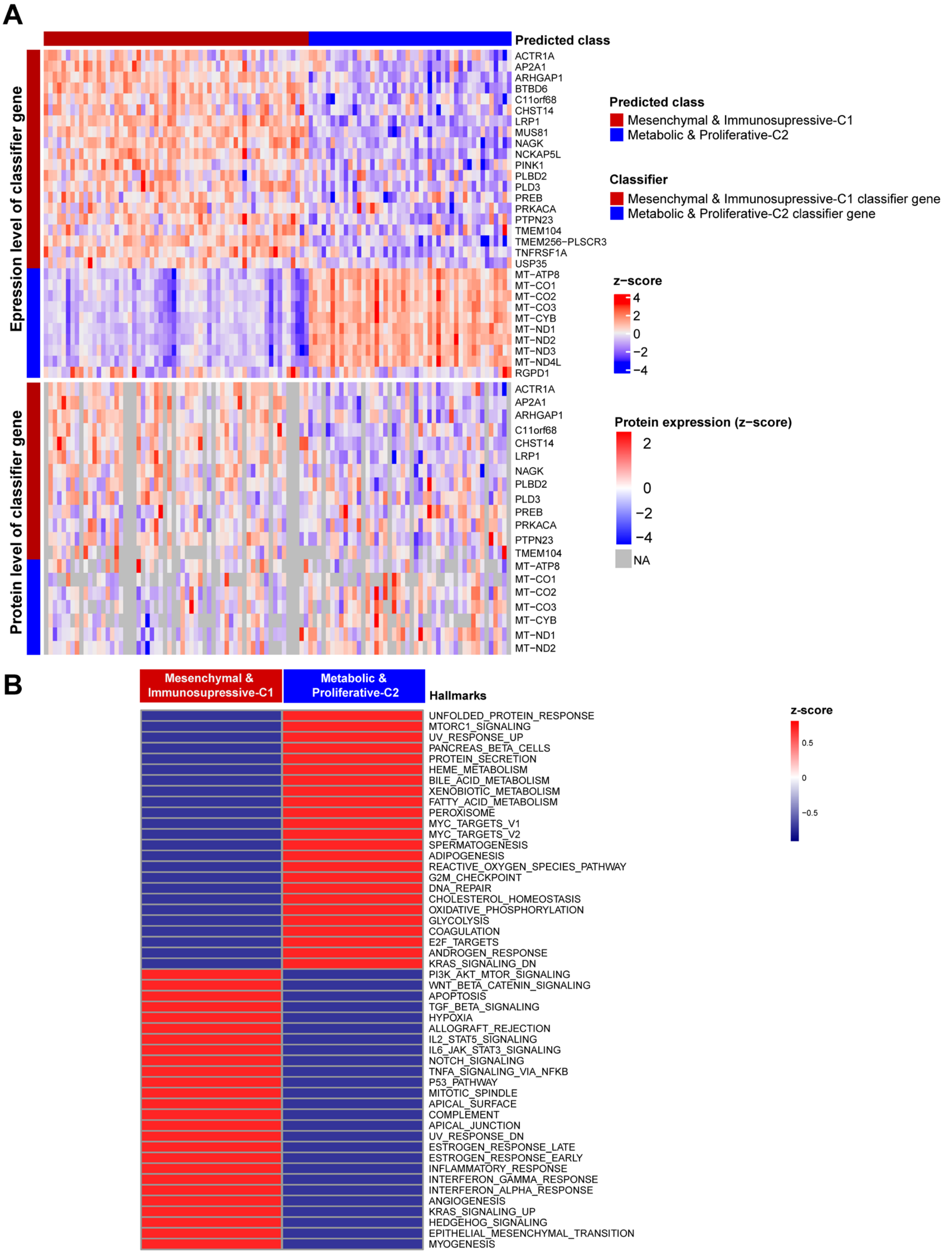

Supplementary Fig. 11. Validation of classifier and molecular classification scheme on the Dong cohort. (**A**) Heatmap representing the RNA and protein expression levels of classifier genes in each sample (Dong cohort, N=255 iCCAs). The RNA and protein expression levels were

represented by normalized z-scores. The molecular class was predicted by nearest template prediction (NTP) analysis. Grey color represents unavailable data (NA). (**B**) Heatmap representing the enrichment of hallmark gene sets in molecular classes for the Dong cohort. Single-sample gene set enrichment analysis (ssGSEA) was used to obtain enrichment scores, with samples from the same subtype indicated with a normalized z-score.

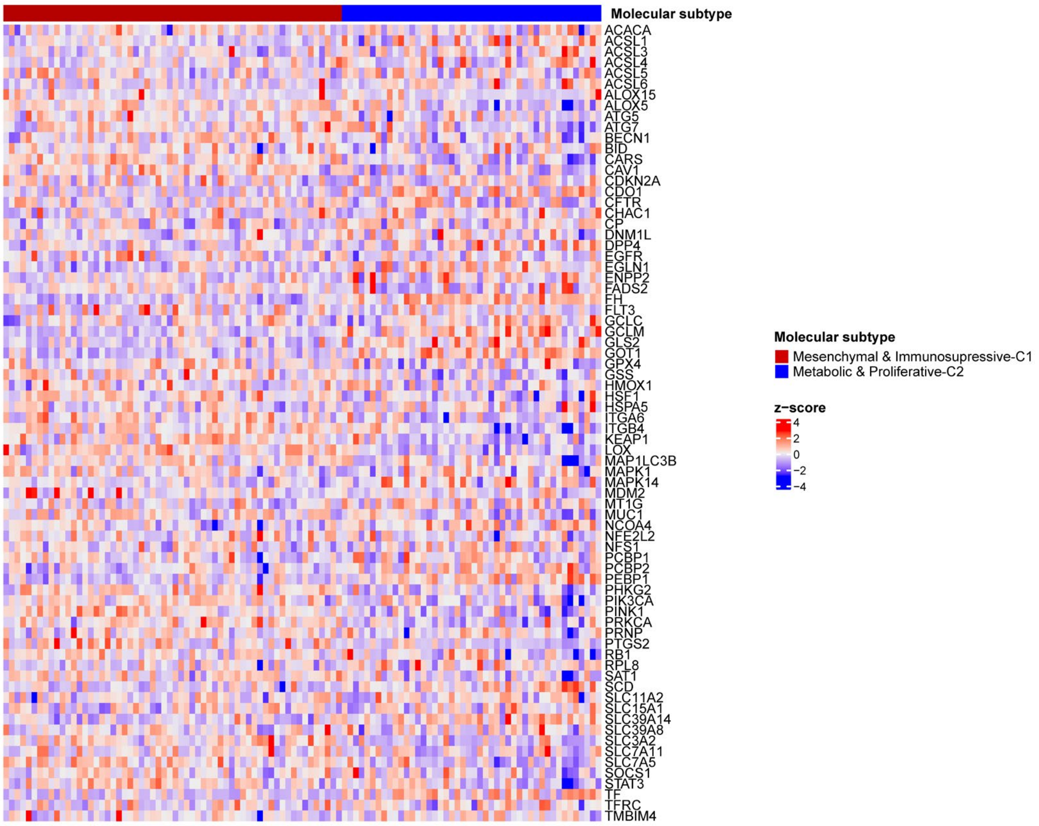

Supplementary Fig. 12. Validation of ferroptosis-related features of molecular classes on the Dong cohort. Heatmap displaying expression levels of ferroptosis-related genes in each sample. The expression values were normalized and represented by z-scores.

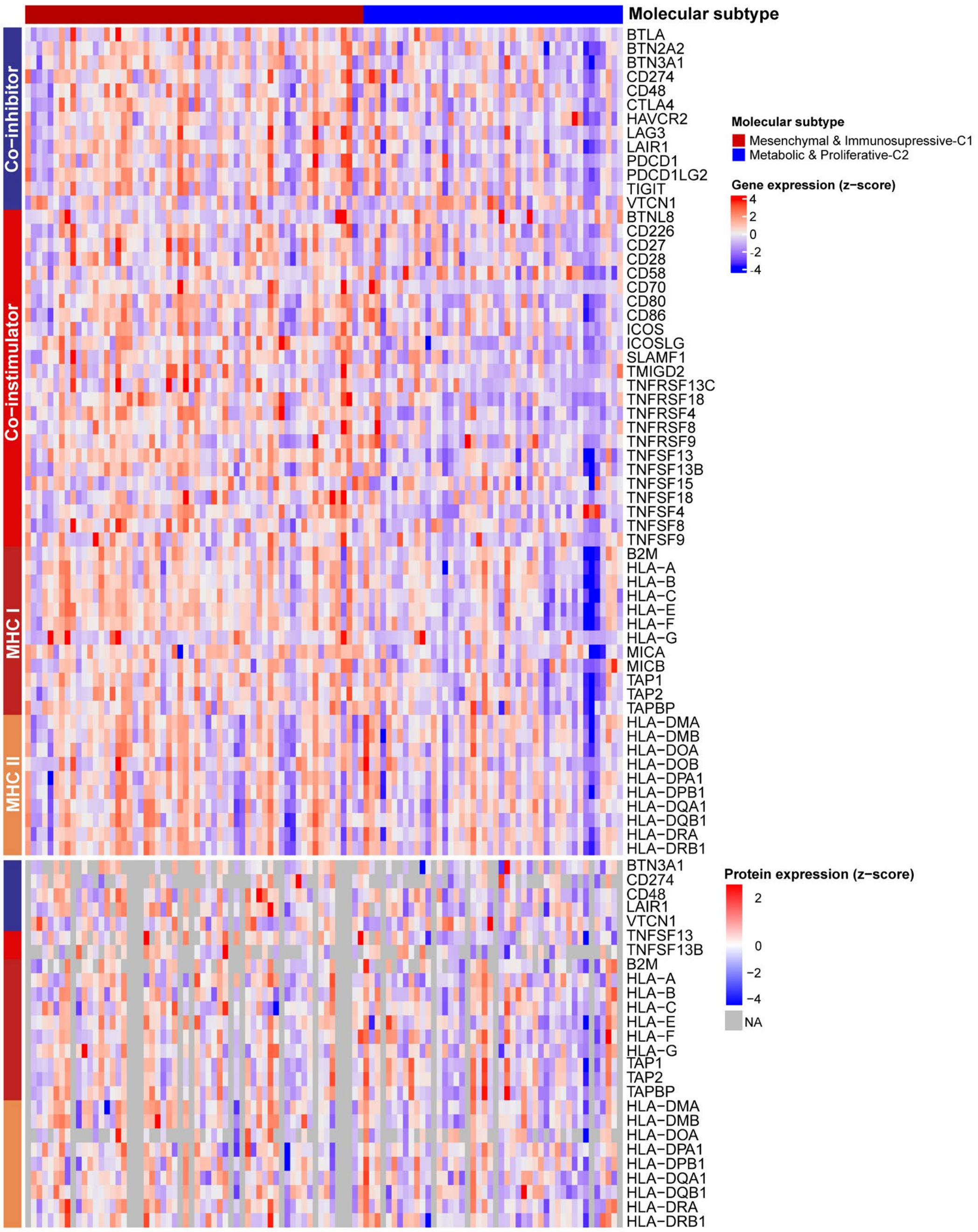

Supplementary Fig. 13. Validation of immune escape mechanisms of molecular classes on the Dong cohort. Heatmap displaying RNA and protein expression levels of genes encoding co-stimulators, co-inhibitors and MHC antigens in each molecular class. The expression values were

normalized and represented by z-scores. Grey color represents unavailable data (NA).

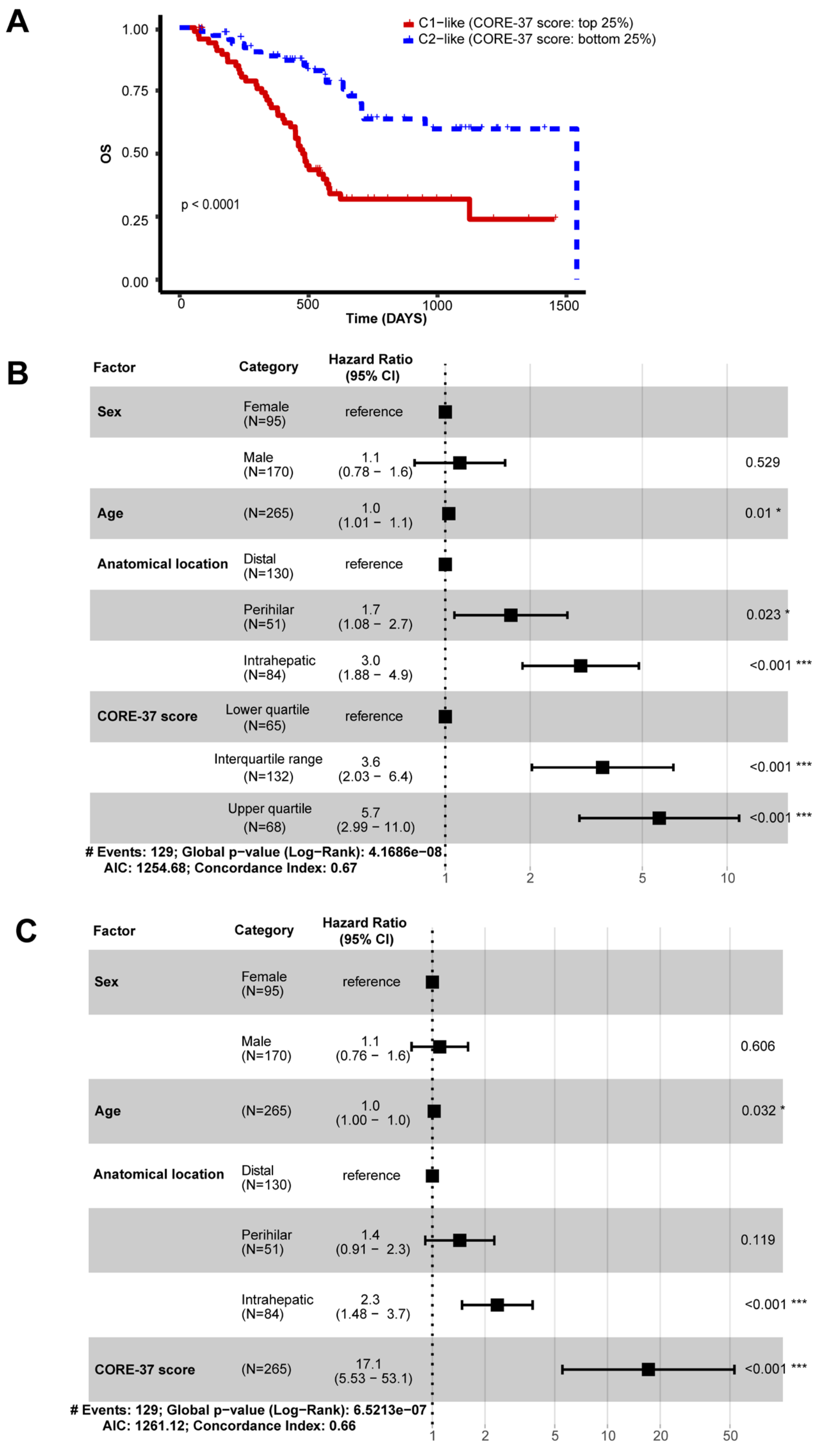

Supplementary Fig. 14. Validation of the developed prognostic indicator on the verification cohort. (**A**) Kaplan-Meier curve comparing overall survival (OS) between samples within the top and bottom quartiles of total scores. Forest plots illustrating (**B**) quartile-categorized total score and (**C**)

continuous total score as independent prognostic indicators, regardless of age, sex and anatomical location. N=265. *P*-values were calculated by log-rank test. CI: confidence interval.

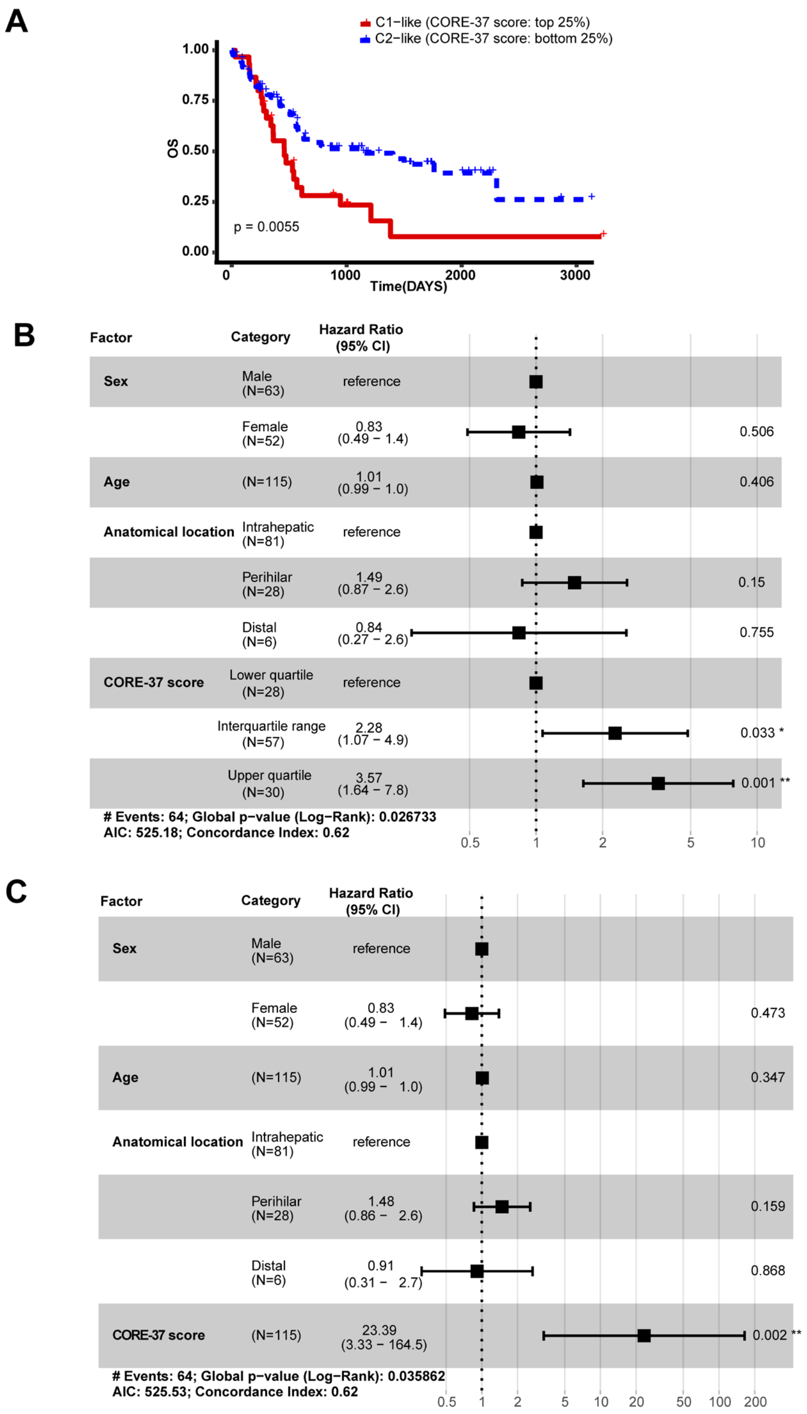

Supplementary Fig. 15. Validation of the developed prognostic indicator on the Jusakul cohort. (**A**) Kaplan-Meier curve comparing overall survival (OS) between samples within the top and bottom quartiles of total scores. Forest plots illustrating (**B**) quartile-categorized total score and (**C**) continuous

total score as independent prognostic indicators, regardless of age, sex and anatomical location. N=115. *P*-values were calculated by log-rank test. CI: confidence interval.

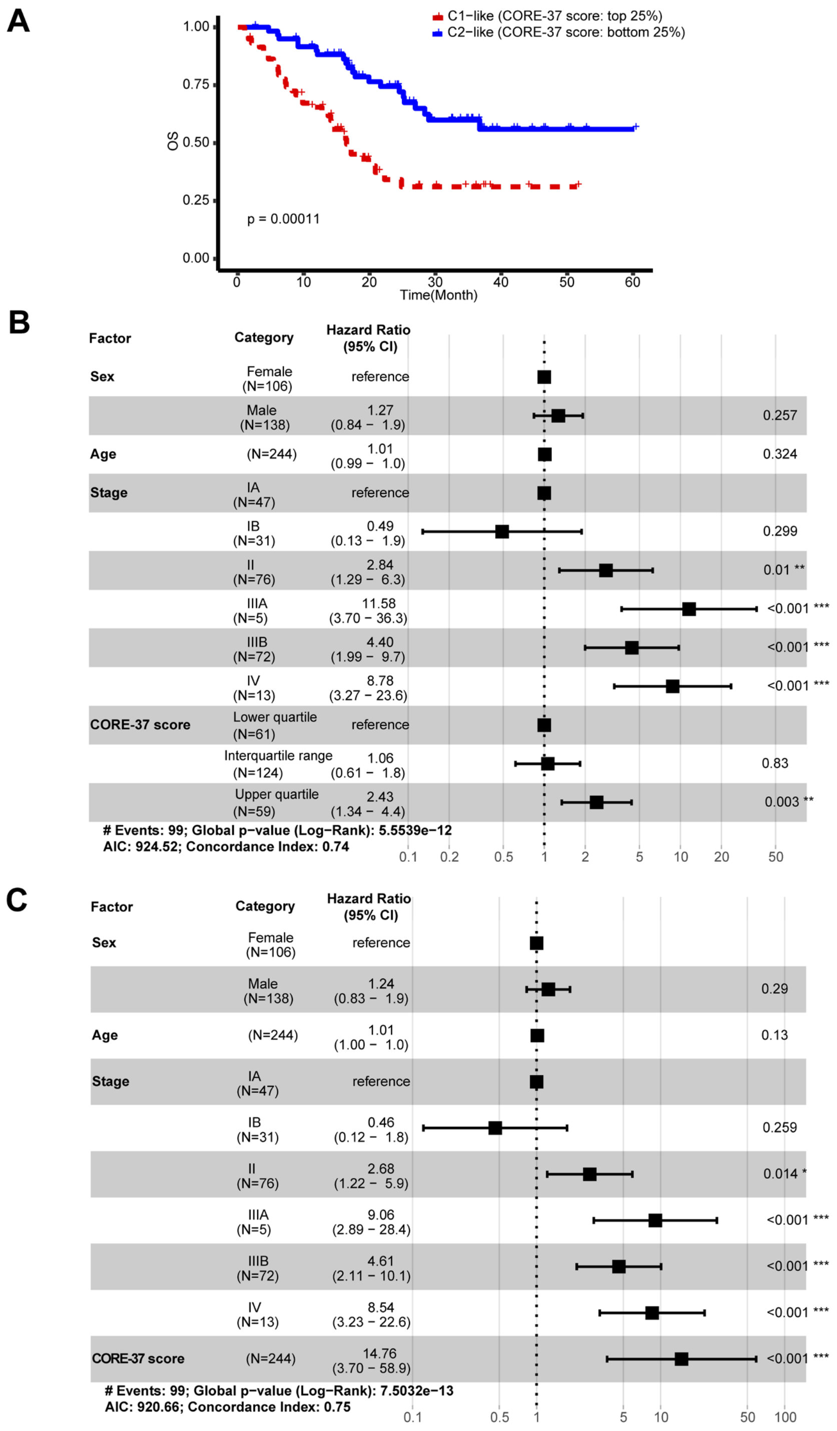

Supplementary Fig. 16. Validation of the developed prognostic indicator on the Dong cohort. (**A**) Kaplan-Meier curve comparing overall survival (OS) between samples within the top and bottom quartiles of total scores. Forest plots illustrating (**B**) quartile-categorized total score and (**C**) continuous total

score as independent prognostic indicators, regardless of age, sex and stage. N=244. *P*-values were calculated by log-rank test. CI: confidence interval.

Supplementary Table 1. Clinical characteristics of CCA patients in the original cohort and stratified cohorts. Summary for the clinical information of patients or samples included in this study. Purified cohort included the remaining samples after filtrating out samples based on NTP results and overall contamination proportions (verification cohort; **see Material and Methods).**

See the EXCEL file attached.

Supplementary Table 2. Clinical information of 438 samples employed in this study.

See the EXCEL file attached.

172 **Supplementary Table 3. Correlation between CCA molecular subtypes**

173 **and clinical and pathological features (N=438).**

**Correlation**	**Statistical method**	**p-value**
Molecular cluster & Sex	Fisher's Exact Test (two-sided)	0.8
Molecular cluster & Resection	Fisher's Exact Test (two-sided)	**0.0001**
Molecular cluster & Stage	Fisher's Exact Test (two-sided)	**1.00E-07**
Molecular cluster & Differentiation	Fisher's Exact Test (two-sided)	0.4335
Molecular cluster & Pathological type	Fisher's Exact Test (two-sided)	0.4
Molecular cluster & Anatomical site	Fisher's Exact Test (two-sided)	**1.00E-06**
Molecular cluster & Age	Kruskal-Wallis rank sum test	**0.006**
Molecular cluster & Hepatic contamination	Kruskal-Wallis rank sum test	**2.20E-16**
Molecular cluster & Pancreatic contamination	Kruskal-Wallis rank sum test	**2.20E-16**
Molecular cluster & Duodenal contamination	Kruskal-Wallis rank sum test	**4.20E-06**
Molecular cluster & Lymphatic contamination	Kruskal-Wallis rank sum test	**0.0036**
Molecular cluster & Neural contamination	Kruskal-Wallis rank sum test	**0.0017**

N=438; $p<0.05$ was considered significant and highlighted with **bold** style.

Supplementary Table 4. Liver-specific and pancreas-specific gene markers as templates for NTP analysis.

See the EXCEL file attached.

Genes were collected at Tissue-specific Gene DataBase in cancer (TissGDB: https://bioinfo.uth.edu/TissGDB/).

Supplementary Table 5. Correlation between CCA molecular subtypes and clinical and pathological features on the purified cohort (N=164).

**Correlation**	**Statistical method**	**p-value**
molecular cluster & Sex	Fisher's exact test (two-sided)	0.59
molecular cluster & Resection	Fisher's exact test (two-sided)	0.97
molecular cluster & Stage	Fisher's exact test (two-sided)	**0.03**
molecular cluster & Differentiation degree	Fisher's exact test (two-sided)	0.36
molecular cluster & Pathological type	Fisher's exact test (two-sided)	0.28
molecular cluster & Anatomical location	Fisher's exact test (two-sided)	0.12
molecular cluster & Age	Kruskal-Wallis rank sum test	0.55
molecular cluster & Liver percentage	Kruskal-Wallis rank sum test	**0.02**
molecular cluster & Pancreas percentage	Kruskal-Wallis rank sum test	0.47
molecular cluster & Duodenum percentage	Kruskal-Wallis rank sum test	NA
molecular cluster & Lymphoid percentage	Kruskal-Wallis rank sum test	0.54
molecular cluster & Neuron percentage	Kruskal-Wallis rank sum test	0.20

N=164; $p<0.05$ was considered significant and highlighted with **bold** style.

Supplementary Table 6. Genes selected for the developed molecular classifier.

See the EXCEL file attached.

Class Neighbors tool from GenePattern web was used to identify classifier genes. These were selected based on the dot plot representing signal-to-noise ratio (SNR) score versus gene rank (**Fig. 5B**).

Supplementary Table 7. The results of NTP analysis.

See the EXCEL file attached.

Supplementary Table 8. Differentially expressed genes (DEGs) selected for estimating the prognostic biomarker “Total Score” in each sample.

See the EXCEL file attached.

A total of 37 DGEs were involved in the construction of prognostic biomarker, with the application of “Singscore” R package. The *p* values are two-sided and $p<0.05$ was considered significant. The $\log_2$ fold change values and adjusted *p*-values (p-adj) for each gene were listed. N=164.

Supplementary Table 9. Net reclassification index comparison between different models.

Model		control=age + TNM-staging			control=age + TNM-staging		
		new=age+ CORE-37			new= age+ TNM-staging+ CORE-37		
**Anatomical location**	Item	Estimate	Lower	Upper	Estimate	Lower	Upper
**dCCA**	NRI	0.171	-0.234	0.550	0.248	-0.031	0.551
	NRI+	0.109	-0.078	0.281	0.146	-0.014	0.291
	NRI-	0.062	-0.193	0.331	0.102	-0.086	0.360
	Pr(Up|Case)	0.366	0.163	0.524	0.341	0.090	0.495
	Pr(Down|Case)	0.257	0.122	0.395	0.195	0.000	0.364
	Pr(Down|Ctrl)	0.331	0.070	0.530	0.302	0.012	0.563
	Pr(Up|Ctrl)	0.269	0.147	0.359	0.199	0.021	0.252
**pCCA**	NRI	0.230	-0.174	0.551	0.196	-0.022	0.630
	NRI+	0.120	-0.133	0.313	0.085	-0.059	0.376
	NRI-	0.111	-0.116	0.271	0.110	-0.013	0.310
	Pr(Up|Case)	0.277	0.017	0.495	0.243	0.000	0.535
	Pr(Down|Case)	0.158	0.016	0.398	0.158	0.000	0.267
	Pr(Down|Ctrl)	0.294	0.044	0.524	0.308	0.000	0.540
	Pr(Up|Ctrl)	0.183	0.027	0.362	0.198	0.000	0.291
**iCCA**	NRI	0.386	-0.168	0.971	0.690	0.185	1.143
	NRI+	0.128	-0.090	0.413	0.274	-0.013	0.514
	NRI-	0.258	-0.160	0.619	0.416	0.153	0.705
	Pr(Up|Case)	0.500	0.393	0.652	0.501	0.259	0.706
	Pr(Down|Case)	0.372	0.191	0.496	0.227	0.149	0.359
	Pr(Down|Ctrl)	0.476	0.305	0.731	0.553	0.277	0.792
	Pr(Up|Ctrl)	0.218	0.083	0.491	0.137	0.055	0.226

NRI: net reclassification index; Pr: proportion; Ctrl: control; dCCA: distal cholangiocarcinoma; pCCA: perihilar cholangiocarcinoma; iCCA: intrahepatic cholangiocarcinoma.

Supplementary Table 10. The raw TPM expression data of 438 samples employed in this study.

See the EXCEL file attached.
