# Supplementary material for: A transcriptome based molecular classification scheme for cholangiocarcinoma and subtype-derived prognostic biomarker
Source: Nat Commun. 2024 Jan 11;15:484. doi: 10.1038/s41467-024-44748-8 (PMC10784309; doi:10.1038/s41467-024-44748-8)
Supplement: Supplementary file 3 — Reporting Summary [file 41467_2024_44748_MOESM3_ESM.pdf]

nature portfolio

Corresponding author(s): NCOMMS-23-24237

Last updated by author(s): Jun 8, 2023

Reporting Summary

Nature Portfolio wishes to improve the reproducibility of the work that we publish. This form provides structure for consistency and transparency in reporting. For further information on Nature Portfolio policies, see our Editorial Policies and the Editorial Policy Checklist.

Statistics

For all statistical analyses, confirm that the following items are present in the figure legend, table legend, main text, or Methods section.

n/a	Confirmed	
☐	☒	The exact sample size (n) for each experimental group/condition, given as a discrete number and unit of measurement
☐	☒	A statement on whether measurements were taken from distinct samples or whether the same sample was measured repeatedly
☐	☒	The statistical test(s) used AND whether they are one- or two-sided<br>*Only common tests should be described solely by name; describe more complex techniques in the Methods section.*
☐	☒	A description of all covariates tested
☐	☒	A description of any assumptions or corrections, such as tests of normality and adjustment for multiple comparisons
☐	☒	A full description of the statistical parameters including central tendency (e.g. means) or other basic estimates (e.g. regression coefficient) AND variation (e.g. standard deviation) or associated estimates of uncertainty (e.g. confidence intervals)
☐	☒	For null hypothesis testing, the test statistic (e.g. F, t, r) with confidence intervals, effect sizes, degrees of freedom and P value noted<br>*Give P values as exact values whenever suitable.*
☐	☒	For Bayesian analysis, information on the choice of priors and Markov chain Monte Carlo settings
☐	☒	For hierarchical and complex designs, identification of the appropriate level for tests and full reporting of outcomes
☐	☒	Estimates of effect sizes (e.g. Cohen's d, Pearson's r), indicating how they were calculated

Our web collection on statistics for biologists contains articles on many of the points above.

Software and code

Policy information about availability of computer code

Data collection	A total of 438 patients with bile duct cancer including iCCA, pCCA and dCCA, who underwent curative-intent operation between 2016 and 2021 at The First Hospital of Jilin University were involved in this study. Formalin-fixed, whole slide section paraffin-embedded (FFPE) samples were obtained from each patient. Pathological diagnosis and the estimation of non-cancer tissue area /total tissue area ratio (including hepatic, pancreatic, duodenal, lymphatic and neural tissues contamination) for each sample were done by two independent pathologists. This study was conducted in accordance with the Declaration of Helsinki and was approved by the Institutional Review Boards of The First Hospital of Jilin University (No: 21Q023-001). Informed consent was obtained from all subjects involved in the study. The clinical information of patients in this cohort is provided in Supplementary Materials.<br>Total RNA was successfully extracted from 438 FFPE samples using TRIzol and RNeasy MinElute Cleanup Kit (Invitrogen). RNA purity was assessed using the NanoDrop Spectrophotometer (Thermo Fisher Scientific™, Waltham, USA). RNA integrity and concentration were measured with the RNA Nano 6000 Assay Kit of the Bioanalyzer 2100 system (Agilent Technologies, Palo Alto, CA, USA). Subsequently, mRNA libraries were created by using the NEB Next® UltraTM RNA Library Prep Kit (NEB, Beverly, MA, USA), following the manufacturer's protocol. Geneplus-2000 sequencing platform (Geneplus, Beijing, China) was utilized to sequence the constructed RNA-seq libraries. The sequencing reads containing adaptor sequences and low-quality reads were removed to obtain high-quality reads. Reads passing quality control were aligned to the human genome hs37d5 using STAR software. Transcript assembly was conducted by using StringTie2.<br><br>Our constructed classifier and molecular classification scheme were tested using two external datasets: the transcript per million (TPM) matrix and clinical information from 36 patients in the TCGA-CHOL project (dCCA=2, pCCA=4, iCCA=30) were downloaded at https://www.cbioportal.org; the TPM matrix and clinical information from 255 patients in the Dong cohort, as well as the protein expression matrix available for 214 patients, were downloaded.<br>The Dong dataset was also used for prognostic biomarker validation. Additionally, the clinical information and expression data available for 115 patients in the Jusakul cohort (iCCA=81, pCCA=28, dCCA=6) were downloaded for validating biomarker.

Data analysis

Nearest template prediction (NTP) analysis was performed with R package CMScaller version 2.0.1 to determine samples showed liver-specific or pancreas-specific expression pattern. Samples with FDR <=0.1 in NTP result and with an overall tissue contamination proportion >25% were selected into the "verification cohort", while the other samples selected into the "purified cohort".
Non-negative matrix factorization (R package NMF version 0.24.0) and consensus clustering method (R package ConsensusClusterPlus version 1.58.0) were applied to obtain unsupervised molecular classes from the original and purified cohorts, respectively. To construct expression-based dendrogram, the Euclidean distance between samples was calculated and hierarchical cluster analysis was performed with the "average" cluster method.
Gene set variation analysis was performed using R package GSVA version 1.42.0 , with method='ssgsea' for 50 hallmark gene sets from MSigDB collections and hypoxia-related, and method='zscore' for ferroptosis-related gene sets.
To investigate the tumor microenvironment, stromal and immune scores were calculated using R package ESTIMATE version 1.0.13. The CIBERSORTx web tool (https://cibersortx.stanford.edu/) was applied to estimate the abundances of interested immune cells of interest in the tumor milieu. Additionally, the Tumor Immune Dysfunction and Exclusion (TIDE) web tool (http://tide.dfci.harvard.edu/) was utilized to estimate the exclusion of infiltrating CD8+ cytotoxic T cells.
To construct a molecular classifier, the Class Neighbors tool from the GenePattern web (https://cloud.genepattern.org/) was applied to identify genes that were closely correlated with molecular class templates. Based on the impact of genes on signal-to-noise ratio (SNR) scores, genes defining each molecular class were selected to construct the classifier, which was further tested on validation cohorts with the application of NTP module from the GenePattern web (https://cloud.genepattern.org/)
To construct subtype-derived prognostic biomarker, R package DESeq2 version 1.34.0 was used for gene differential expression analysis between two molecular classes.
R package singscore version 1.14.0 was applied to compute sample-wise enrichment scores for two signatures, which outputted a unified score for the complete signature (CORE-37 score) as well as scores for C1-like and C2-like signatures separately.
The usefulness of CORE-37 biomarker was validated by receiver operator characteristic (ROC) curve comparison (R package timeROC version 0.4) and net reclassification index (NRI) estimation (R package nricens version 1.6).
The computational analysis, statistical analysis and plot generation in this study were performed using R software version 4.1.2 under the RStudio environment (https://www.r-project.org/).

For manuscripts utilizing custom algorithms or software that are central to the research but not yet described in published literature, software must be made available to editors and reviewers. We strongly encourage code deposition in a community repository (e.g. GitHub). See the Nature Portfolio guidelines for submitting code & software for further information.

Data

Policy information about availability of data

All manuscripts must include a data availability statement. This statement should provide the following information, where applicable:

- Accession codes, unique identifiers, or web links for publicly available datasets
- A description of any restrictions on data availability
- For clinical datasets or third party data, please ensure that the statement adheres to our policy

The raw TPM expression data of 438 samples employed in this study is provided in Supplementary Materials. The source code for bioinformatics analyses is available from the authors upon formal request.

Research involving human participants, their data, or biological material

Policy information about studies with human participants or human data. See also policy information about sex, gender (identity/presentation), and sexual orientation and race, ethnicity and racism.

Reporting on sex and gender	This study enrolled 438 patients, 67.35% of them were male, 32.65% of them were female. Sex was determined based on self-reporting. Those data can be shared of individual-level data.
Reporting on race, ethnicity, or other socially relevant groupings	The socially constructed or socially relevant categorization variables was not used in our study.
Population characteristics	A total of 438 patients with bile duct cancer including iCCA, pCCA and dCCA were involved in this study. All patients are East Asians. The median age of the participants was 63 (range, 25-82), and 67.35% of them were male. With regards to anatomical location, 43.15% were diagnosed with dCCA, 30.82% with pCCA, and 26.03% with iCCA. Pathological TNM stage differed by anatomical locations, with IIA/IIB in dCCA, II/IIIC in pCCA, and II/IIIB in iCCA.
Recruitment	A total of 438 patients with bile duct cancer including iCCA, pCCA and dCCA, who underwent curative-intent operation between 2016 and 2021 at The First Hospital of Jilin University were involved in this study. Informed consent was obtained from all subjects involved in the study.
Ethics oversight	This study was conducted in accordance with the Declaration of Helsinki and was approved by the Institutional Review Boards of The First Hospital of Jilin University (No: 21Q023-001).

Note that full information on the approval of the study protocol must also be provided in the manuscript.

Field-specific reporting

Please select the one below that is the best fit for your research. If you are not sure, read the appropriate sections before making your selection.

☒ Life sciences ☐ Behavioural & social sciences ☐ Ecological, evolutionary & environmental sciences

For a reference copy of the document with all sections, see nature.com/documents/nr-reporting-summary-flat.pdf

Life sciences study design

All studies must disclose on these points even when the disclosure is negative.

Sample size	A total of 438 patients with bile duct cancer including iCCA, pCCA and dCCA, who underwent curative-intent operation between 2016 and 2021 at The First Hospital of Jilin University were involved in this study. We have obtained transcriptomic data of 438 clinically-annotated CCA tumors with different anatomical sites, which to our knowledge is the largest cohort investigating CCA molecular classification at transcriptomic level.
Data exclusions	The transcriptome data of all samples pass the quality inspection and need not be excluded
Replication	All replication attempts were successful
Randomization	Given that hepatic and pancreatic contamination were the most predominant in our cohort, we collected previously published liver-specific and pancreas-specific gene markers as templates for NTP analysis (Table S3). After filtrating out samples based on NTP results and overall contamination proportions (see Material and Methods), we retained a total of 164 samples which is referred to as the "purified cohort" for the subsequent analyses, while the other 274 samples were used as the "verification cohort" (Fig. 1B, Table 1).
Blinding	The persons performing sample preparation and sequencing were unaware of the sample identity. All data have been documented and are available upon reasonable request

Reporting for specific materials, systems and methods

We require information from authors about some types of materials, experimental systems and methods used in many studies. Here, indicate whether each material, system or method listed is relevant to your study. If you are not sure if a list item applies to your research, read the appropriate section before selecting a response.

Materials & experimental systems

n/a	Involved in the study	
☒	☐	Antibodies
☒	☐	Eukaryotic cell lines
☒	☐	Palaeontology and archaeology
☒	☐	Animals and other organisms
☒	☐	Clinical data
☒	☐	Dual use research of concern
☒	☐	Plants

Methods

n/a	Involved in the study	
☒	☐	ChIP-seq
☒	☐	Flow cytometry
☒	☐	MRI-based neuroimaging
