# Supplementary material for: A transcriptome based molecular classification scheme for cholangiocarcinoma and subtype-derived prognostic biomarker
Source: Nat Commun. 2024 Jan 11;15:484. doi: 10.1038/s41467-024-44748-8 (PMC10784309; doi:10.1038/s41467-024-44748-8)
Supplement: Supplementary file 4 — Description of Additional Supplementary Files [file 41467_2024_44748_MOESM4_ESM.docx]

**Description of Additional Supplementary Files**

Supplementary Data 1

Description: The Supplementary Data 1 includes the tables covered in the Supplementary Information, including clinical characteristics of CCA patients, the raw TPM expression data of 438 samples employed in this study, liver-specific and pancreas-specific gene markers for NTP analysis and other data supporting the findings of this study.

Supplementary Movie 1

Description: No Supplementary Movie are included in this article

Supplementary Software 1

Description: No Supplementary Software are included in this article
